# Communal Cookery

**Published:** 1917-09-22

**Authors:** 


### COMMUNAL COOKERY.

### A New Opportunity for Educated Women.

By A SOCIAL WORKER.

After the nursery, the kitchen. After the call to women to *Save the Babies*, a call to save the physical efficiency of the growing children—indeed, of the whole nation—by right use of material. The problem has for some time been before us, but one aspect of it has been brought under special notice by very recent events. First, Mrs. Lloyd George's letter in the *Times* demanding recruits for domestic science schools, and pleading that other work, much of it temporary for the duration of the war, should not interfere with this most important of careers for women. Following on this, letters from disappointed parents, who say that they have spent money in training girls vainly since teachers' posts are not numerous enough, and letters from girls corroborating the statement. At the same moment the papers are full of such developments as the newly opened communal kitchen at Hammersmith, which will, when its fifteen distributing centres are in working order, send out some 30,000 meals daily.

In all parts of the country local authorities, Food Control Committees, and other bodies are starting, or considering, their own ventures. Even the rural districts have awakened to a sense of their possibilities. In the present rapid moving of events these first efforts may develop, almost before we can realise what is happening, into a vast undertaking carried out all over the country, requiring for its administration a large number of skilled workers, and offering to such workers excellent prospects on an ascending scale. Here is—in substantiation of Mrs. Lloyd George's statement, and as a reply to those who have been discouraged by what seems to have been a dark hour before dawn—a definite offer from the highest authority, that of the Ministry of Food.

### The Food Ministry Offers Free Training.

The Ministry of Food will now receive three candidates at a time for three weeks' training. They must be able to produce the diploma of a recognised school of cookery, or otherwise to satisfy the constituted authority of fitness to receive this specialised instruction. They will receive payment at the rate of 35s. weekly and their meals. Application should be made to the Ministry of Food, Grosvenor House, London, W. 1.

Students will be taught, besides the actual routine of the kitchen and the distribution of food, the necessary items of storekeeping and bookkeeping on the large scale required, catering estimates, etc., and, of course, all those important lessons which have been formulated by past experience and continue to develop. The posts likely to be first available are those of sub-organisers and organisers, probably for the Ministry itself, and for the various Councils, Food Control Committees, and school authorities, municipalities, and whatever other bodies may take up the work of communal or co-operative kitchens. Besides these, there will very soon be openings in the management of canteens for camps and factories—the latter a new development that has come to stay—and for colleges, large institutions, and great hotels. The system described by a cook of the old type a little while since as "plenty of stuff going anyhow and nohow" is not one which is likely to thrive unchecked in the future.

### Wasteful Conditions.

War-time rises in the cost of all kinds of food have made prosperous housekeepers consider their ways, while they have poignantly affected those who have always had to look twice at every sixpence spent. We have realised suddenly the wastefulness in material, firing, and labour of our subdivided families in the middle and upper classes. Among the poor we have realised the greater proportionate loss caused by buying in small quantities, missing always the profitable purchase of the "cut and come again" or the "four for 3½d." order. We must add the frequent ignorance of method and the physical exhaustion of the mother, who may have to fit her catering and cookery into the leisure (!) left by a day's charing, or may have to prepare and serve meals in one living-room with—not impossibly—the care of an infant or a case of sickness. The resulting dreary monotony of food, largely bread and "relishes" of tinned provisions, has too often the effect of failing to nourish the children, and creating in the elders a craving for stimulants. War conditions have made the problem less acute in some households, in so far that they have removed the father or big brothers who needed most meat, and left the mother with a dependably regular income. On the other hand, not only have food prices doubled, but the increased cost of such necessaries for the family as boots involves some stinting.

### The Communal Kitchen Come to Stay.

At the same time one of the topsy-turvy results of the war has been to send a number of young gentlewomen into military kitchens to cook under strict discipline, and for hours which no trade union would tolerate. However temporary their service, it will leave its mark in eyes opened to many things hitherto unthought of and unnoticed. Finally, the conditions of domestic service have reached suddenly the goal to which they had long been tending. "As merry as the maids" is an old-time proverb belonging to days when large houses and house parties were the rule, with a correspondingly large domestic staff. A generation brought up to consider amusement and society essential features of life will not readily confine itself to the cottage or flat when "the long rubber of domestic life" below stairs is played by cook and house-parlourmaid only. The great house, the club, the hospital, and all those service flats and boarding houses which group a fortuitous concourse of human beings into a new type of family will no doubt long continue to staff themselves without much difficulty. But as regards the provision of meals, we may safely prophesy that the communal kitchen has come to stay, and will, in time, cater for all classes, and that it will before long be followed by some form of common dining-room.

The most Utopian ideas seem thus to be brought suddenly within the scope of practical politics. We may see the cheerful social meal depicted by William Morris in his "Dream of John Ball" actually realised. We may even see in existence the new town-planning described a quarter of a century ago by the American enthusiast, Mrs. Perkins Gilman, where the artisan's wife, having taken her children to the school and her washing to the laundry attached to her block of grouped

(Continued on page 518.)

### COMMUNAL COOKERY—(*concluded from p.* 516).

dwellings, should tidy her home, its solid cleansing being done at intervals by professional cleaners, and sit down to read till the time for a common dinner in the appropriate hall.

### An Experiment in Hampshire.

Meantime an interesting practical experiment is working in the rural district of Christchurch in Hampshire, where seven out of the eleven villages comprised have joined in a communal scheme for children's dinners at school. On any of the five working days of the week, fifty to sixty boys and girls may be seen seated at the long school desks in one of these villages, a long strip of white American cloth being laid down and plates placed on it. Whereas formerly many of these children munched bread in the playground or went home to a dinner of it, thick soups, stews, milk puddings, and cooked fruit are provided. In this particular village children under seven pay 1d., seven to nine pay 1½d., and over nine pay 2d., and for this sum have two courses, soup and pudding or stew and pudding, with second helpings if desired, but no bread. The saving on bread in these seven schools, estimated modestly at 6 oz. per head per day, works out at 2,844½ lb. in four weeks. The gain in weight of the children has been very satisfactory, and the plan, varied in different villages according to local conditions, is self-supporting and involves no charity. This particular village sends off, in a market cart, dinners for thirty children in a small hamlet 1½ miles away, another economy. Gifts of fruit and vegetables have, however, been included, and a varying service of some voluntary helpers. These items, however, may well be set against the serious rise in cost of many provisions, and have also a further usefulness, in that they interest farmers and tradesmen in the great national question of children's nourishment. Beyond this, a very valuable work must be done in introducing new and economical and nourishing kinds of food into village menus.

### A Much Neglected Food.

Soup, for instance, the common food of the Continental child, is sadly seldom made, or made decently, even in lower middle-class families. The country doctor may hesitate to recommend it because beef-tea, a stimulant rather than a food, is its best known form. The thick, well-skimmed and well-cooked mixture of meat juice and vegetables, with perhaps a little dumpling or bit of oaten or maize bannock is admirably nourishing. If rice pudding—often a novelty also—and well-stewed fruit follow, it is not surprising if children, lured on by other children, have occasionally converted a parent from hesitation to unqualified approval. The sympathy and practical help of teachers throughout the experiment is no small testimony to its value.

Altogether there is urgent need for the super-cook.

